# Flexible Embedded Metal Meshes by Nanosphere Lithography for Very Low Sheet Resistance Transparent Electrodes, Joule Heating, and Electromagnetic Interference Shielding

**DOI:** 10.1021/acsaelm.5c00425

**Published:** 2025-04-28

**Authors:** Mehdi Zarei, Khashayar Mohammadi, Abdullah A Mahmood, Mingxuan Li, Paul W. Leu

**Affiliations:** † Department of Mechanical Engineering, University of Pittsburgh, Pittsburgh, Pennsylvania 15261, United States; ‡ Department of Civil Engineering, University of Waterloo, Waterloo N2L3G1, Canada; § Department of Electrical and Computer Engineering, University of Pittsburgh, Pittsburgh, Pennsylvania 15261, United States; ∥ Department of Chemical Engineering, University of Pittsburgh, Pittsburgh, Pennsylvania 15261, United States; ⊥ Department of Industrial Engineering, University of Pittsburgh, Pittsburgh, Pennsylvania 15261, United States

**Keywords:** EMI shielding, reactive ion etching, nanosphere lithography, metal mesh, flexible transparent conductive electrode, joule heating

## Abstract

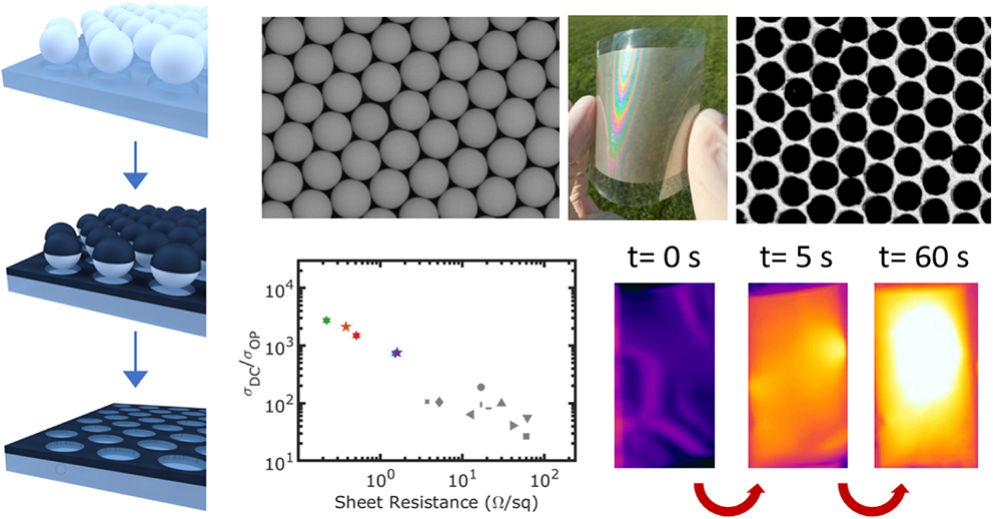

We demonstrate the highest transparent electrode performance among metal meshes fabricated via nanosphere lithography (NSL), achieving an order-of-magnitude improvement in the figure of merit FoM (σ_*DC*_/σ_*OP*_). Additionally, we present, for the first time, the application of metal meshes fabricated by NSL for transparent electromagnetic interference (EMI) shielding, enabled by exceptional improvements in sheet resistance. Our NSL method produces substrate-embedded metal meshes in PET and glass by etching trenches, yielding high-aspect-ratio features with low sheet resistance. Embedded structures also exhibit superior robustness during bending and tape tests compared to sputtered metallic films on the surface. As a transparent electrode, the flexible Ag meshes exhibit a sheet resistance of 1.52 Ω/sq and transparency of 73.1% as well as a sheet resistance of 0.22 Ω/sq and transparency of 58.1%, corresponding to FoMs of 737 and 2736, respectively. For transparent EMI shielding, the flexible metal meshes achieve a shielding efficiency (SE) of 34.5 dB with 73.1% visible transmission and an EMI SE of 52.8 dB with 58.1% visible transmission. As a flexible heater, the metal meshes can reach a saturation temperature exceeding 70^◦^C within 60 s under an applied voltage of 1.2 V. These embedded metal meshes hold promise for applications requiring ultralow sheet resistance, including heated windows and defrosting systems, large-area organic light-emitting diode (OLED) lighting and displays, solar cells, and EMI shielding.

## Introduction

Indium tin oxide (ITO) is the most widely used transparent conductive electrode (TCE) material, but it struggles to meet modern optoelectronic demands for functionality, flexibility, and affordability. Indium is a scarce and expensive metal, and ITO’s brittle nature makes it prone to cracking under mechanical stress, leading to deteriorated electrical performance and potential device failure. Furthermore, its compatibility with flexible substrates remains limited. Polymer-based substrates necessitate lower processing temperatures, reducing ITO crystallinity and increasing sheet resistance. To replace ITO, metal networks, including metal meshes and metal nanowires, have been extensively studied as TCEs due to their high transparency and low sheet resistance.^1−7^ This has led to significant interest in using these materials for transparent electromagnetic interference (EMI) shielding. With our increasing reliance on electronic devices and systems, there is a growing demand for effective EMI shielding materials that protect electronic components from radiation damage and block unwanted signals.^8−15^

In response to the need for EMI shielding, a broad range of materials have been investigated, including metal films^8,9,16−19^ metal meshes^2,20^ metal nanowires^21−24^ graphene^25^ carbon nanotubes^26,27^ and MXenes.^28,29^ Moreover, many modern optoelectronic devices, such as light-emitting diodes (LEDs), car windows, displays, touchscreens, solar cells, and optical communication systems, require materials that are not only transparent but also capable of blocking radio frequency (RF) signals.^30^ Achieving this balance, however, remains a significant challenge. Metal nanowires have challenges due to their nonuniform distribution, inherent percolation limitations, and significant contact resistances between wires. In contrast, metal meshes tend to maintain consist uniform properties without percolation and contact issues. Numerous established techniques have been devised to fabricate metal meshes, encompassing methodologies like photolithography^31,32^ imprint lithography^33^ 3D printing approaches^34,35^ crack lithography^20,36−39^ and electrodeposition.^40^ Nevertheless, the majority of these technologies tend to be costly, time-intensive, and often unsuitable for large-scale production.^41,42^ Photolithography, for example, is a widely used method for creating uniform, small-width patterns. However, its application may not be well-suited for large-scale applications due to its reliance on costly equipment and cleanroom conditions. 3D printing techniques such as inkjet, gravure, screen, and flexography are typically constrained by relatively poor resolution, with minimum achievable line widths generally exceeding 20 μm. Another common technique involves using a prefabricated mold to transfer patterns onto a flexible substrate. However, this technique faces its own challenges, such as issues with removing the mold, ensuring conformal contact, and creating complex features.

Reactive metal inks have gained significant attention in recent years for fabricating metal meshes due to their capability to produce high-aspect-ratio metal structures using various lithography techniques or 3D printing,^31,32,36^ Additionally, their low curing temperature makes them compatible with flexible substrates.^43^ Our group recently developed a novel sputter-free fabrication method that utilizes reactive silver ink to fill high-aspect-ratio embedded trenches in PET, significantly improving the performance of metal meshes produced by this technique.^36^ However, the inherent randomness of crack formation and the limited control over pitch and width can restrict the ability to design custom mesh patterns optimized for specific optical or electronic properties.

Nanosphere lithography (NSL) has been the focus of extensive research in recent years due to its straightforward and easy fabrication process.^44−61^ Additionally, its ability to control pitch and width by simply adjusting the size of the microspheres, combined with the capability to fabricate the sphere mask directly on the substrate without requiring any cleanroom equipment, is a significant advantage. However, the primary drawback of this method lies in its limited performance as a transparent electrode, with its ratio of direct current conductivity to optical conductivity (σ_*DC*_/σ_*OP*_) figure of merit rarely exceeding 100.^45^ This metric is a standard for assessing the efficiency of transparent conductive electrodes, where a higher value indicates a device capable of transmitting more electrical current while maintaining excellent optical transparency. In contrast, alternative approaches such as photolithography, crack lithography, imprint lithography, or 3D printing can readily achieve σ_*DC*_/σ_*OP*_ figures of merit exceeding 1000.^45,62^

To fabricate metal meshes using this method, most studies have deposited metal after plasma treatment to form a conductive metallic network on the substrate. Yet, the thickness achieved through this method is typically limited to only 50 nm for liftoff,^45^ resulting in suboptimal electrical performance. While electrodeposition can improve sheet resistance after fabricating metal meshes, its isotropic nature may lead to a reduction in transmission. Furthermore, since conductive metals such as silver (Ag) and copper (Cu) tend to adhere poorly to substrate surfaces, it becomes necessary to use an expensive adhesive layer.^45^ Despite the addition of such adhesive layers, achieving robust mechanical performance, including successful bending and tape tests, remains a challenge for this method.^45^

In this paper, we provide the first demonstration of a flexible embedded meal mesh by NSL and its application to TCEs and EMI shielding. We employ a facile fabrication method to transfer the NSL patterns to both PET and glass substrates. Using inductively coupled plasma reactive ion etching (ICP-RIE), high aspect ratio trenches were created and then filled with metal. Unlike previous approaches, where metal meshes were directly deposited onto substrates with limited thickness, our embedded meshes enable significantly higher aspect ratio features, leading to very low sheet resistance under 1.6 Ω/square and the highest σ_*DC*_/σ_*OP*_ figures of merit for NSL approaches. Embedded structures also exhibit superior robustness during bending and tape tests compared to sputtered metallic films on the surface. Our flexible embedded Ag meshes achieve sheet resistance (*R*_*s*_) of 1.52 and 73.1% visible transmission, which corresponds to a σ_*DC*_/σ_*OP*_ figure of merit of 737. These meshes also achieve *R*_*s*_ = 0.22 Ω/sq and 58.1% visible transmission, which corresponds to σ_*DC*_/σ_*OP*_ = 2736. For transparent EMI shielding, the samples exhibit a shielding efficiency (SE) of 34.5 dB with 73.1% visible transmission and an SE of 52.8 with 58.1% visible transmission. In glass, the meshes achieve a sheet resistance of 1.61 Ω/sq and a transparency of 75.1% (σ_*DC*_/σ_*OP*_ of 756) and an EMI SE of 36.3 dB. Our findings exceed the performance of previous NSL studies in the literature as TCEs by an order of magnitude. Furthermore, we present the first demonstration of EMI shielding using metal meshes fabricated through NSL, achieved by improving the sheet resistance. Serving as a flexible heater, the metal meshes can reach saturation temperatures of up to 70 °C within 60 s when supplied with 1.2 V. The integration of metal meshes into PET offers several advantages for different large-area applications, including heated windows and defrost systems, large-area organic LEDs^63−65^ solar cells^66^ and other optoelectronic devices.

## Results and Discussion

**Figure 1:**
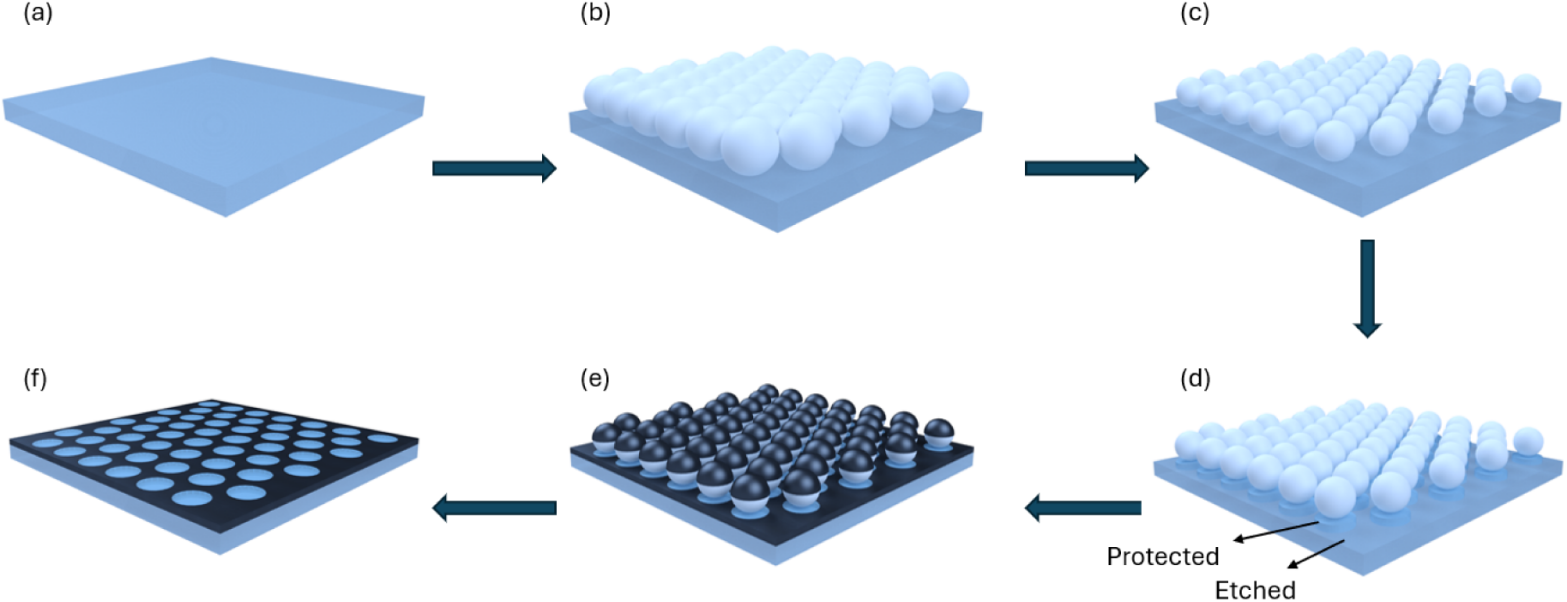
Schematic of the substrate-embedded Ag mesh fabrication process: (a) transparent flexible PET or rigid glass substrate, (b) deposition of a monolayer of microspheres on the substrate, (c) O_2_ plasma treatment, (d) inductively coupled plasma reactive ion etching (ICP-RIE), (e) electron-beam evaporation of Ag, (f) removal of microspheres by a liftoff process, resulting in exposed Ag-embedded metal meshes.

Figure 1 presents the schematic of the fabrication process. A transparent, flexible PET or rigid glass substrate is cleaned (Figure 1a), and a monolayer of hexagonally close packed 3 μm polystyrene (PS) microspheres is transferred onto the substrate via self-assembly at the air/water interface (Figure 1b). A schematic of the nanosphere lithography process is provided in Figure S1. The Marangoni effect drives the spreading of microspheres at the water/air interface and a sodium dodecyl sulfate (SDS) surfactant is used to reduce surface tension and pack the microspheres together at the interface. Additional details can be found in the Experimental section and the Supplemental Text. Then, plasma treatment via inductively coupled plasma reactive ion etching (ICP-RIE) is used to reduce the microspheres’ size (Figure 1c). The size of the microspheres determines the pitch size in the metal nanomesh. All samples in this work utilize 3 μm PS microspheres. ICP-RIE is applied to the substrate to etch the spaces between the microspheres into the substrate (Figure 1d). Because ICP-RIE is anisotropic and proceeds primarily in the vertical direction, the substrate area directly beneath the microspheres remains unetched (as indicated by the arrow denoting the protected region in Figure 1d), whereas the unprotected areas undergo etching. This vertical-only etching is crucial for preserving the substrate regions covered by the microspheres and ensuring a well-defined transfer of the microsphere-patterned gaps. While the ICP-RIE tool is well-suited for high-power etching processes, we observed that using excessively high power caused extra etching of the microspheres and the substrate (Figure S2). Then, silver (Ag) is deposited onto the substrate (Figure 1e). Finally, by removing the microspheres, a substrate-embedded Ag mesh is achieved (Figure 1f).

Plasma treatment time and ICP-RIE time are key fabrication parameters that can be adjusted to produce metal mesh samples with varying hole diameters and metal mesh thicknesses. Plasma treatment is commonly employed to reduce the size of microspheres by etching, where reactive species in the plasma selectively remove material from the surface.^44^ The degree of shrinkage depends on several factors, including gas composition, power, pressure, and treatment duration. In our process, we carefully optimized these parameters to achieve controlled and consistent size reduction of the microspheres. There is no inherent limitation to the size reduction, as extended plasma treatment can further decrease their diameter, allowing for additional refinement if needed. In this study, the pitch is consistently maintained at 3 μm due to the use of 3 μm diameter microspheres. Table 1 provides details of the five samples fabricated on both PET and glass. By selecting different ICP-RIE times, varying thicknesses were achieved for the five samples, as summarized in Table 1. The etch times for the five samples were 3, 3, 7, 12, and 20 min, respectively. The thicknesses of the five samples were measured using cross-sectional SEM images, and were 350, 350, 800, 550, and 900 nm, respectively. For samples 4 and 5, the trench depth was directly measured from the cross-sectional SEM images provided in Figure 4c. For PET substrates, significant charging was observed in metal-filled samples, making direct measurement challenging. Instead, the trench depth was estimated by multiplying the etch rate by the etch time. The etch rate was determined by etching a sample, removing the microspheres, and acquiring a cross-sectional SEM image, as shown in Figure S3. The measured etch rate for PET substrates was approximately 1.9 nm/s.

**Table 1:** Summary of Sample Types and Fabrication Parameters

Sample number	Substrate	Plasma time (s)	ICP-RIE time (min)	Thickness (nm)	Hole diameter (μm)
1	PET	80	3	350	2.8
2	PET	105	3	350	2.6
3	PET	120	7	800	2.5
4	Glass	80	12	550	2.8
5	Glass	90	20	900	2.6

Conventional metal meshes typically fabricated using NSL are formed by the deposition of metals onto the substrate. However, this method is constrained by thickness limitations due to liftoff issues that occur when the metal layer becomes too thick. In contrast, our approach overcomes these limitations by embedding the metal meshes into the substrate, enabling the creation of thicker, high-aspect-ratio structures.

**Figure 2:**
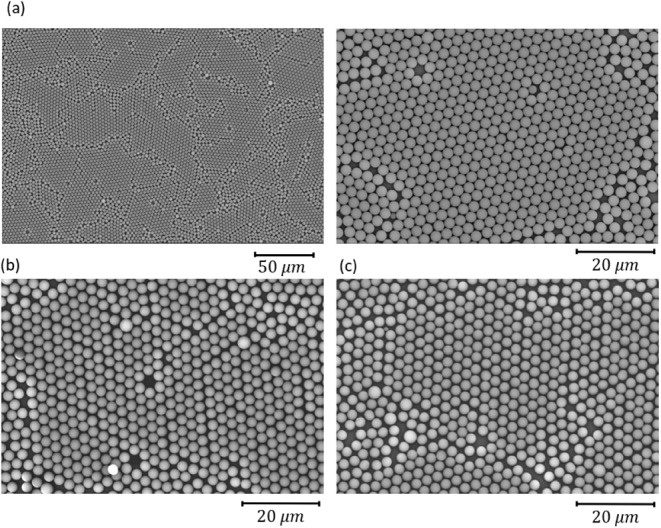
SEM images of (a) a monolayer of 3 μm microspheres on PET at two different magnifications, (b) after 80 s of O_2_ plasma treatment, (c) after ICP-RIE.

Figure 2 presents SEM images of the PS microspheres on the substrate at different stages of the fabrication process. Figure 2a shows SEM images of a monolayer of 3 μm PS microspheres on a PET substrate at two different magnifications. The order of the resulting microparticle array primarily depends on the quality of the colloidal monolayer. Nonuniformities and cracks in the formation of a homogeneous microsphere arrangement are evident in the SEM images. These additional cracks are attributed to the lateral shrinkage of the colloidal spheres relative to the solid support substrate during the drying process.^67^ Unfortunately, such cracks are an inherent aspect of this process and inevitably degrade the order of the resulting nanoparticle array.^67^ Lateral shrinkage of colloidal spheres during the drying process occurs due to capillary forces as the liquid evaporates, resulting in surface tension and drying-induced stresses that cause compression and shrinkage. This shrinkage can induce mechanical stress and irregular cracking in the monolayer. To mitigate this, advanced drying methods have been extensively employed to improve monolayer formation and prevent cracking in the dried film.^67^ Furthermore, larger microsphere sizes tend to result in higher nonuniformity and more pronounced cracking due to their larger variations in diameter. The use of 3 μm microspheres, as demonstrated in this work, has been rarely reported in the literature. In Figure 2b, plasma treatment reduced the size of the microspheres. Following this, ICP-RIE was applied to the substrate, and an SEM image was captured while the microspheres remained on the substrate, as shown in Figure 2c. A comparison of (b) and (c) reveals that ICP-RIE has a negligible effect on the microsphere size, enabling the formation of deep trenches. It should be noted that the overall shrinkage of the microspheres is due to both the plasma and ICP-RIE processes. The gaps between the microspheres were transferred onto the substrate, after which the microspheres were removed. SEM images of the resulting substrate, captured in top-view and side-view, are presented in Figure S3a,b, respectively. While this step is not part of the fabrication process, as outlined in Figure 1, the microspheres were removed here to clearly visualize the trenches formed in the substrate. After shrinking the microspheres by etching, Ag was deposited, and the microspheres were removed, resulting in embedded metal meshes within the substrate.

**Figure 3:**
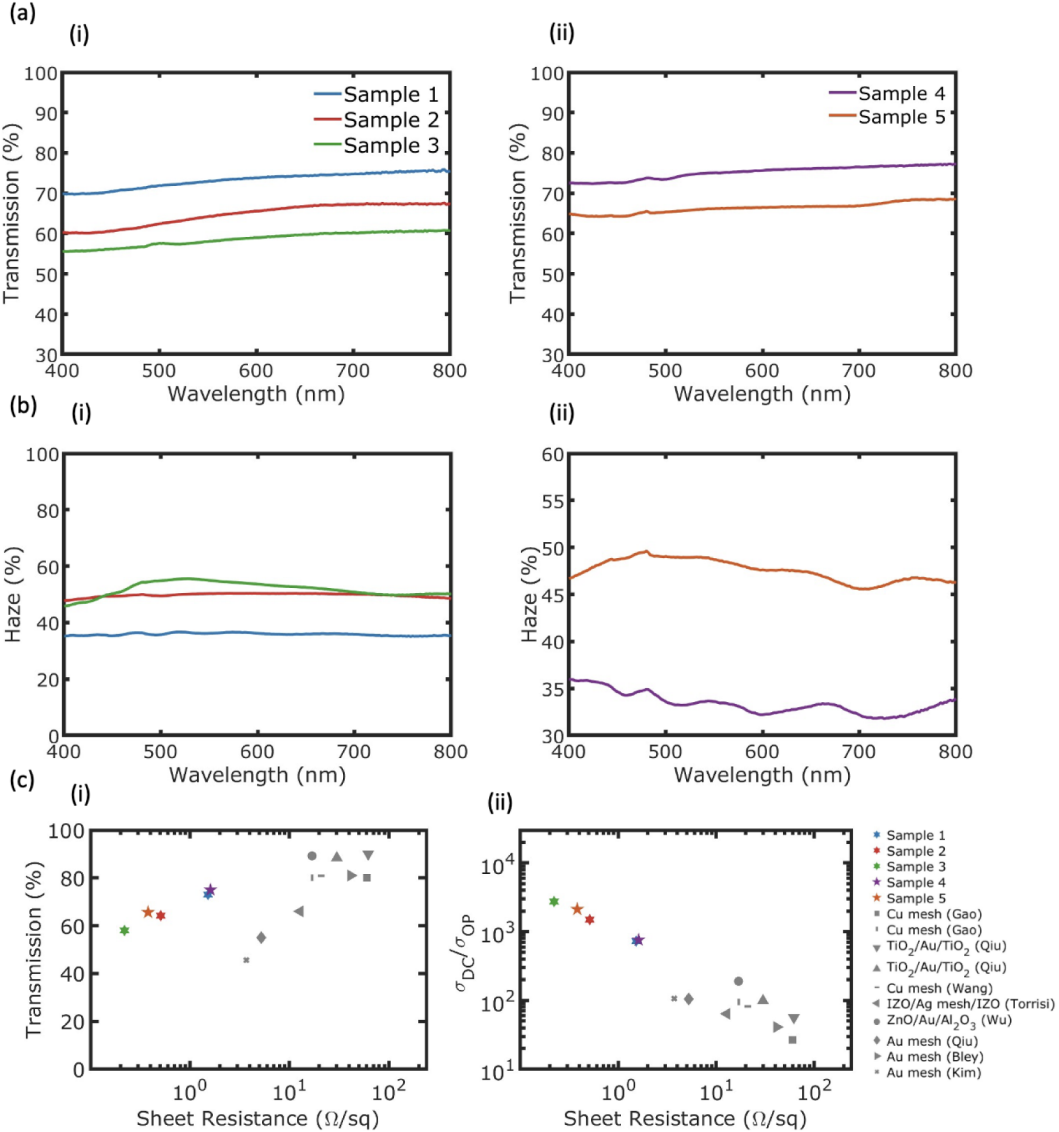
Optical performance of the five Ag mesh samples: (a) transmission as a function of wavelength within the visible spectrum, (b) haze as a function of wavelength within the visible spectrum, and (c) transparent electrode performance of Ag mesh samples from this work compared to other metal meshes fabricated via NSL reported in the literature. (i) Transmission at 550 nm versus sheet resistance and (ii) σ_DC_/σ_OP_ versus sheet resistance. The literature works are summarized in Table S1.

Figure 3 presents the optical properties, including (a) transmission and (b) haze, of the fabricated (i) PET metal meshes and (ii) glass metal meshes. The transmission values have been adjusted to exclude the influence of the substrate. As shown, the transmission of both PET and glass metal mesh samples remains relatively flat across the visible wavelength range, with no resonance observed. This is because the scale of the metal meshes does not correspond to the wavelength scale of visible light. Metal meshes typically exhibit a fairly flat transmission profile throughout the visible wavelength range.^31,32,36,68^ Samples 1 to 5 possess transmissions of 73.1%, 64.3%, 58.1%, 75.1%, and 65.6%, respectively, at 550 nm. The transmission of metal meshes produced via NSL typically remains below 85% for two main reasons. First, the size of the interstices in a hexagonally close-packed colloidal monolayer is determined by the diameter of the colloidal spheres, which is approximately 10%. Second, as the liquid evaporates, irregular cracks develop due to lateral shrinkage of the colloidal spheres relative to the solid support, ultimately compromising the nanoparticle array’s ordering. A basic geometric model can be used to qualitatively predict the average theoretical total transmission *T*_th_ of a metal nanomesh film with hexagonally ordered circular perforations, based on the corresponding void fraction. The metal fraction is assumed to be completely nontransmitting due to reflection and absorption within the metal, while the holes are considered fully transmitting. This model neglects plasmonic and photonic effects, as well as imperfections in the 2D ordering of the nanomesh:
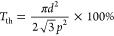
1

Calculating *T*_th_ for the fabricated samples based on *p* = 3 μm and the measured hole diameter *d* yields transmission values of 79.0%, 68.1%, 63.0%, 79.0%, and 68.6% for Samples 1–5, respectively. A comparison between the experimental and theoretical transmission values is summarized in Table 3. The discrepancies between the theoretical and experimental values can likely be attributed to the nonuniform arrangement of the microspheres.

Figure 3b shows the haze behavior of the samples. Samples 1–5 achieve haze values of 36.2%, 50.3%, 55.0%, 33.6%, and 48.7%, respectively, at 550 nm. While samples 1 and 2 have the same thickness, the higher haze achieved for Sample 2 can be attributed to its smaller hole diameter. Sample 3, with deeper trenches and a smaller hole diameter compared to Sample 2, exhibits even higher haze. Similarly, sample 5 shows higher haze than Sample 4 due to its smaller hole diameter and deeper trenches. However, the effect of thickness remains unclear. To investigate this, we fabricated a new set of three samples on glass, referred to as samples 6, 7, and 8. The transmission and haze plots across the visible wavelength range are shown in Figure S4a,b, respectively. Plasma and ICP-RIE times were adjusted for the samples to achieve approximately the same transmission while varying their metal mesh thicknesses. The hole diameter of all three samples is approximately 2.7 μm. The thicknesses of samples 6, 7, and 8 are 150, 400, and 900 nm, respectively, resulting in corresponding increases in haze to 36.7%, 40.1%, and 46.3%. This represents the first demonstration of haze evaluation with varying thicknesses for metal mesh samples fabricated through NSL. The detailed information regarding the fabrication and performance of these samples is summarized in Table 2.

**Table 2:** Haze Evaluation with Increasing Metal Mesh Thickness at Constant Transmission

Sample number	Substrate	Plasma time (s)	ICP-RIE time (min)	Thickness (nm)	*T*_exp_ (%)	Haze (%)
Sample 6	Glass	140	3.5	150	68.1	36.7
Sample 7	Glass	120	9.0	400	69.0	40.1
Sample 8	Glass	80	20.0	900	67.6	46.3

Further, these samples achieve very high haze, which has not been previously realized for metal meshes fabricated using NSL.^45^ Some optoelectronic applications require high clarity, such as displays, benefit from a nanomesh optimized for low haze. Conversely, applications that require maximization of the light path length within a photoactive film, such as solar cells, or those needing enhanced wide-angle luminescent efficiency, such as LEDs for lighting, are better served by a nanomesh with high haze. In a previous study, it was found that there is a trade-off between achieving high transmission and high haze in nanomeshes fabricated through NSL.^69^ At a constant pitch, reducing the hole diameter resulted in higher haze, as demonstrated through both simulation and experiment. However, the thickness was fixed at 50 nm in that study. Haze is influenced by all geometric parameters, including pitch, width, and thickness. Additionally, the simulation was conducted for a free-standing metal mesh. There remains a lack of simulation and experimental studies investigating the haze of metal meshes embedded in a substrate, particularly with respect to varying the thickness of the metal meshes. Embedded metal meshes are likely to behave differently compared to free-standing or surface-sputtered metal meshes. The reason is that the scattering of light is also influenced by the interaction between the metal mesh and the embedded dielectric substrate.

By creating deep trenches in the substrate and filling them with Ag, low sheet resistances were achieved. Samples 1–5 exhibit sheet resistances of 1.52, 0.51, 0.22, 1.61, and 0.38 Ω/sq, respectively. In contrast, conventional metal meshes fabricated through NSL have struggled to achieve thicknesses greater than 50 nm. Figure 3c(i) presents the transmission and sheet resistance data for our fabricated samples alongside other transparent metal mesh samples fabricated through NSL reported in the literature (refer to Table 2 of this review paper).^45^ As shown, our novel approach of creating deep meshes improves the sheet resistance by an order of magnitude. Figure 3c(ii) presents the ratio of direct current conductivity to optical conductivity (σ_*DC*_/σ_*OP*_) as a function of sheet resistance, defined by
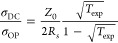
2where *Z*_0_ = 377 Ω is the free space impedance. Our metal mesh samples (1–5) achieve FoMs of 737, 1492, 2736, 756, and 2113, respectively. Compared to other metal meshes fabricated through NSL, which typically struggle to achieve an FoM greater than 100, our method demonstrates an improvement of an order of magnitude. The detailed information on the substrate, experimental transmission, theoretical transmission, haze, *R*_s_ (Ω/sq), and σ_*DC*_/σ_*OP*_ for all the fabricated samples in this work are summarized in Table 3. Further, detailed information about the data points in Figure 3c(ii) is summarized in Table S1. Our findings significantly enhance the performance of metal meshes fabricated through NSL, opening new opportunities to use these structures in various optoelectronic applications, including solar cells, OLEDs, EMI shielding, and more.

**Table 3:** Transparent Electrode Performance of the Five Samples

Literature	Substrate	*T*_exp_ (%)	*T*_th_ (%)	Haze (%)	*R*_*s*_(Ω/sq)	σ_*DC*_/σ_*OP*_
Sample 1	PET	73.1	79.0	36.2	1.52	737
Sample 2	PET	64.3	68.1	50.3	0.51	1492
Sample 3	PET	58.1	63.0	55.0	0.22	2736
Sample 4	Glass	75.1	79.0	33.6	1.61	756
Sample 5	Glass	65.6	68.6	48.7	0.38	2113

**Figure 4:**
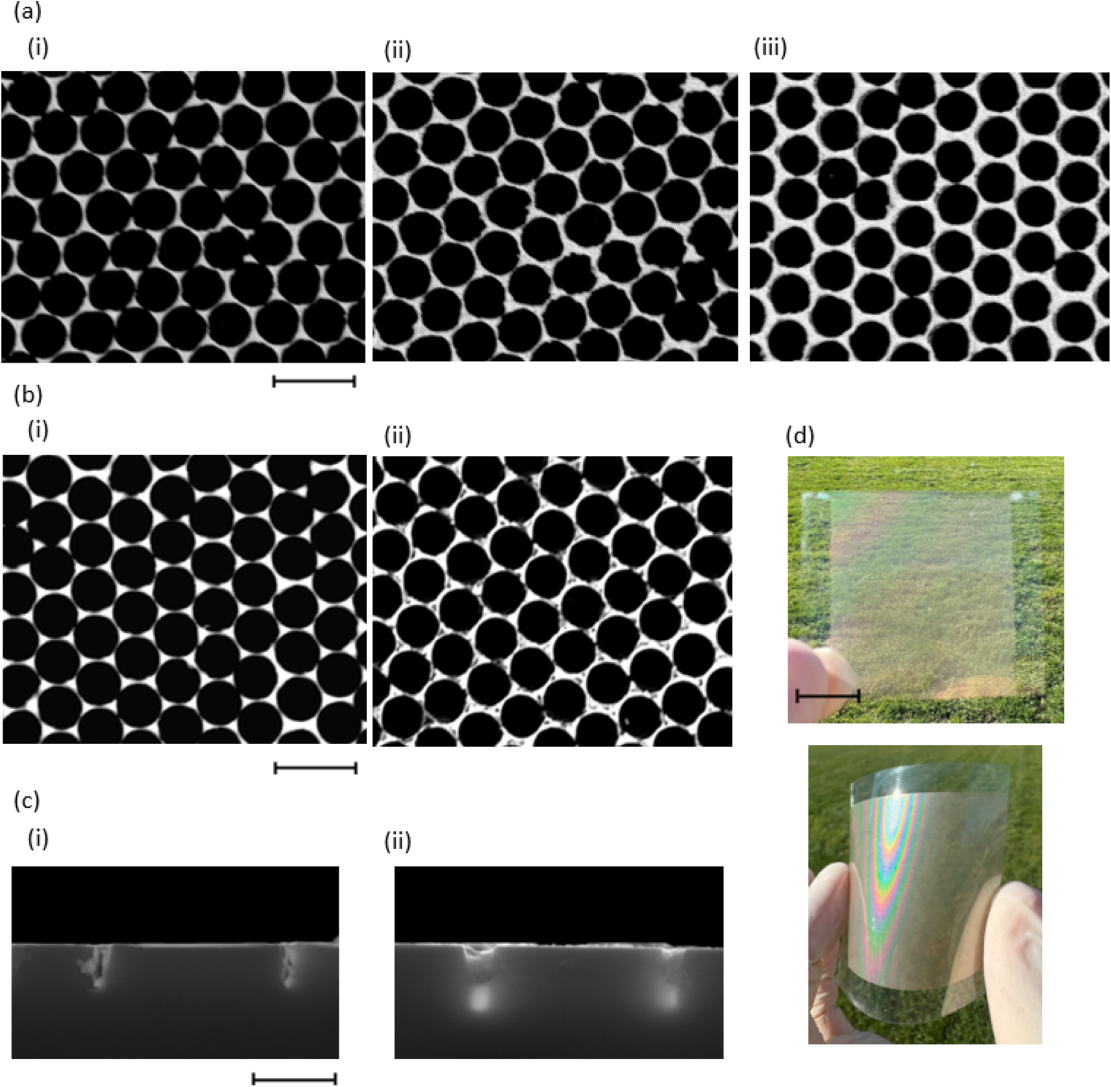
Top-view SEM images of (a) PET-embedded metal meshes: (i) sample 1, (ii) sample 2, (iii) sample 3; and (b) glass-embedded metal meshes: (i) sample 4, (ii) sample 5. (c) Cross-sectional SEM images of: (i) sample 4, (ii) sample 5. The scale bars for (a), (b), (c), and (d) are 5 μm, 5 μm, 1 μm, and 2 cm, respectively. (d) Optical image of sample 1, showing a transmission of 73.1% and haze of 36.2% at 550 nm.

Figure 4 presents top-view SEM images of (a) PET-embedded and (b) glass-embedded metal meshes. In (a), images (i)-(iii) correspond to samples 1, 2, and 3, and in (b), images (i)-(ii) correspond to samples 4 and 5, respectively. The results indicate that increasing plasma treatment time causes higher microsphere shrinkage, resulting in smaller hole diameters. The hole diameters measured through SEM for samples 1, 2, 3, 4, and 5 are 2.8 μm, 2.6 μm, 2.5 μm, 2.8 μm, and 2.6 μm, respectively. We observed that increasing the hole size beyond approximately 2.8 μm leads to inadequate trench filling and poor connectivity in the metal meshes, establishing an upper limit for the hole diameter to achieve good conductivity in the metal meshes. Figure 4c(i) and (ii) show cross-sectional views of samples 4 and 5, respectively. The trenches exhibit a primarily rectangular shape, indicating anisotropic etching with minimal lateral etching. These SEM images demonstrate the effectiveness of the etching recipe in achieving anisotropic etching while preserving the microsphere integrity throughout the process. Further, the deposition of metal films effectively fills the high-aspect-ratio trenches, leading to low sheet resistance.

Figure 4d presents an optical image of sample 1, which exhibits a transmission of 73.1% and a haze of 36.2% at 550 nm. A 1 cm-wide bare PET margin is visible on both the left and right sides of the image, with the metal mesh covering an area of approximately 6 × 7 cm. The image highlights the sample’s optical clarity and high transmission within the visible spectrum, showcasing its suitability for optoelectronic applications. The rainbow color fringes observed in the bottom image are due to the diffraction into various angles by the metal meshes, which is wavelength dependent.

**Figure 5:**
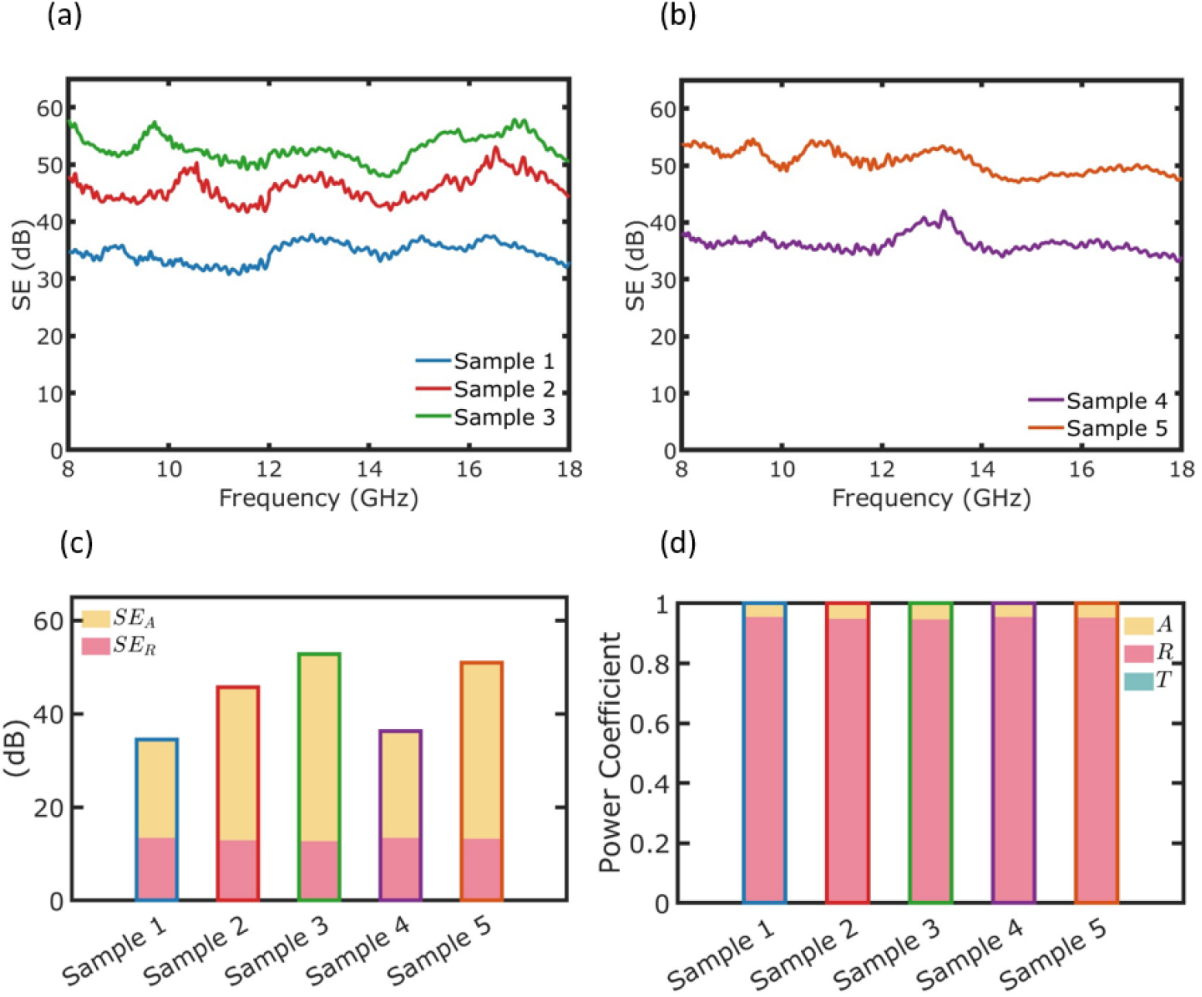
EMI SE vs frequency in the range of 8–18 for (a) PET-embedded and (b) glass-embedded metal meshes (c) SE contribution of SE_*A*_ and SE_*R*_. (d) Power coefficient for five samples.

Figure 5a,b present the EMI shielding effectiveness (SE) of PET-embedded and glass-embedded metal meshes, respectively, as a function of frequency in the range of 8–18 GHz. The SE is calculated using the equation:
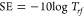
3where *T*_*rf*_ represents the radio frequency transparency within this range. The SE was evaluated across two specific frequency bands: the X-band (8–12 GHz), commonly used in radar systems for air traffic control, weather monitoring, and military applications, and the Ku-band (12–18 GHz), primarily employed in satellite communications. Our five fabricated metal meshes achieved average SE values of 34.5, 45.7, 52.8, 36.3, and 50.9 dB, respectively, across the specified frequency range. Metal meshes typically exhibit consistent EMI SE across the 8–18 GHz range.^32^ The slight variation in the EMI SE of our samples can likely be attributed to nonuniformity in the ordering of the metal meshes, as discussed earlier. A summary of the transparent EMI performance of the five fabricated samples is provided in Table 4.

**Table 4:** Transparent EMI Shielding Performance of the Five Samples

Sample number	*T*_exp_ (%)	SE (dB)
1	73.1	34.5
2	64.3	45.7
3	58.1	52.8
4	75.1	36.3
5	65.6	50.9

Figure 5c provides a detailed analysis of the SE performance of the metal meshes. The total SE comprises two components: reflection shielding effectiveness (SE_*R*_) and absorption shielding effectiveness (SE_*A*_). Refer to the EMI Shielding Measurement section in the Supporting Information for details on these two components of SE. For the five samples tested, the reflection efficiencies are 13.4, 12.9, 12.7, 13.4, and 13.2 dB, respectively, while the absorption efficiencies are significantly higher at 21.1, 32.8, 40.0, 22.8, and 37.6 dB, respectively. Additionally, Figure 5d presents the power coefficients for transmission, reflection, and absorption at radio frequencies for the five samples. The reflection coefficients (*R*_*rf*_) are consistently high across all samples, measured at 0.95, indicating that about 95% of the incident electromagnetic energy is reflected by the mesh. In contrast, the absorption coefficients (*A*_*rf*_) are lower, around 0.05 for all samples, meaning that only about 5% of the energy is absorbed. This confirms that reflection is the primary shielding mechanism. However, despite its smaller contribution, absorption plays an important role by dissipating the portion of energy that is not reflected. The high absorption shielding effectiveness observed indicates that the metal mesh effectively attenuates this residual energy, further enhancing its overall shielding performance.

**Figure 6:**
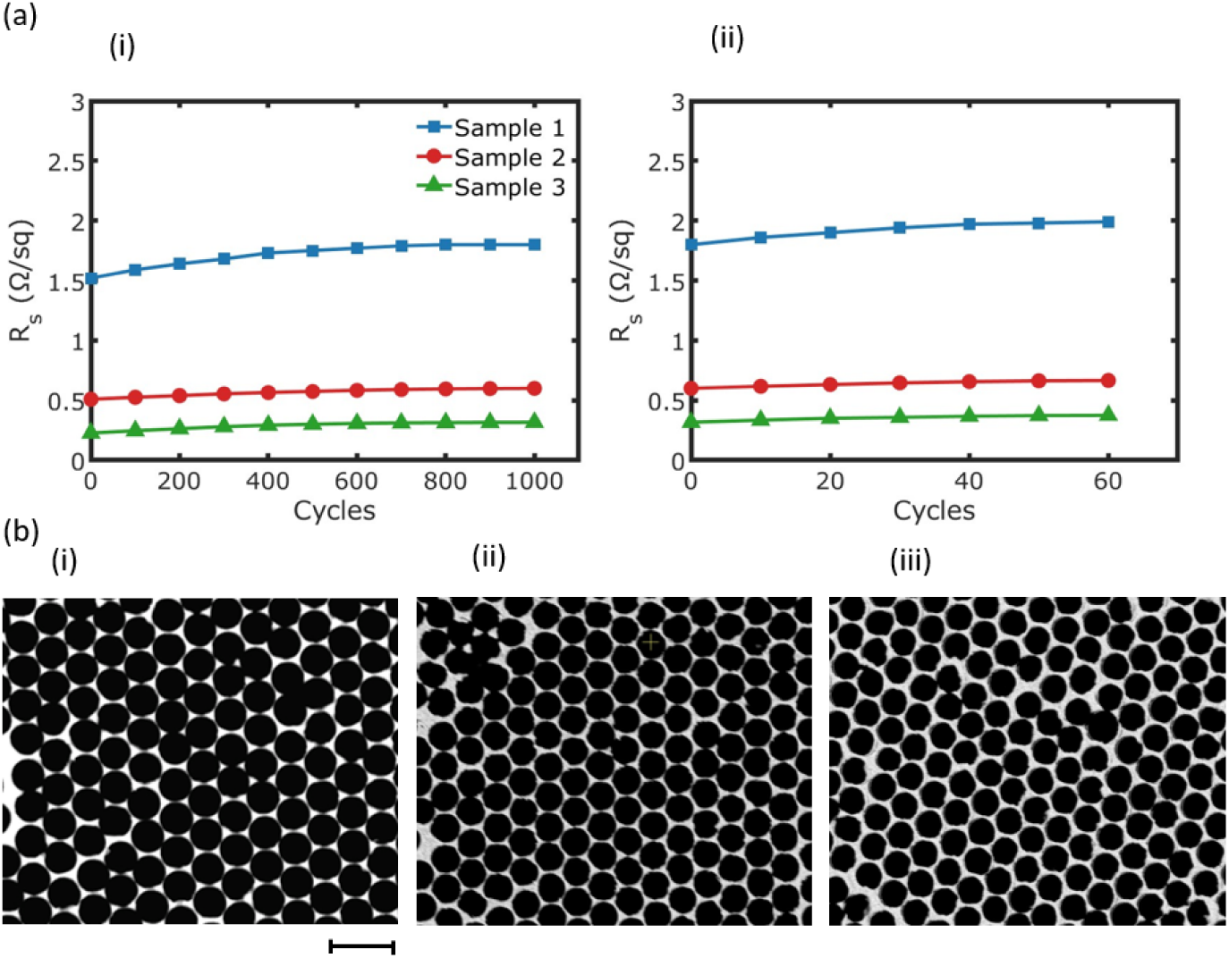
(a) Change in sheet resistance of flexible metal mesh samples during (i) bending test and (ii) tape adhesion test. Note that the tape test was conducted on the same samples following the bending test. (b) SEM images of Samples 1–3, respectively, after both the bending and tape tests. The scale bar shows 5 μm.

To assess the mechanical durability of the embedded metal mesh structures for use in flexible optoelectronics, we performed bending and tape adhesion tests to evaluate the performance and resilience of the fabricated metal meshes. Figures 6a(i) and (ii) illustrate the results of the bending tests and tape adhesion tests, respectively. The sheet resistance was measured after every 100 cycles of bending and is reported as the absolute sheet resistance. The bending radius was set to 4 mm, and the samples were bent under tension. Each cycle involved one instance of bending the sample under tension, followed by releasing the tension. These tests provide insights into the structural integrity and electrical stability of the metal meshes under repeated mechanical stress. During the initial phases of testing, all samples showed a slight increase in sheet resistance. However, as the number of cycles increased, the sheet resistance values eventually stabilized for each sample. After 1000 bending cycles, Samples 1 through 3 exhibited increases in sheet resistance of 0.28, 0.09, and 0.09 Ω/sq, respectively. Following the bending tests, the same samples underwent tape adhesion tests. After 60 adhesion cycles, no significant change in sheet resistance was observed. Samples 1 through 3 displayed increases in sheet resistance of 0.19, 0.07, and 0.06 Ω/sq, respectively. These results highlight the mechanical resilience of the fabricated metal mesh structures, making them suitable for applications requiring flexibility. Although the adhesion of surface-deposited metal meshes fabricated via NSL on PET can be improved by adding a thin layer of Ti, Ni, or Cr, embedding the metal mesh directly into the substrate offers a more effective alternative. This approach enhances durability by protecting the mesh from environmental factors such as corrosion and mechanical stress. SEM images were taken after applying both bending and tape tests to evaluate their effects on the embedded metal meshes (Figure 6b). By comparing these images with those in Figure 4, no significant changes in the shape of the holes or the structure of the meshes are observed. The minimal increase in sheet resistance can likely be attributed to a slight detachment of silver. However, this does not affect the connectivity of the metal meshes or result in any cracking in the film.

The outstanding optoelectronic properties of the Ag mesh film make it a promising candidate for use in transparent electrothermal devices, including applications like thermotherapy and defroster heaters. Joule heating, also known as resistive heating, is the process by which electrical energy is converted into heat when an electric current flows through a resistive material. This effect is described by Joule’s law, given by
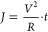
4where *J* represents the heat generated, *V* is the applied voltage, *R* is the resistance of the material, and *t* is the duration of the applied voltage. The equation highlights that the heat produced is directly proportional to the square of the voltage and inversely proportional to the resistance of the material.

**Figure 7:**
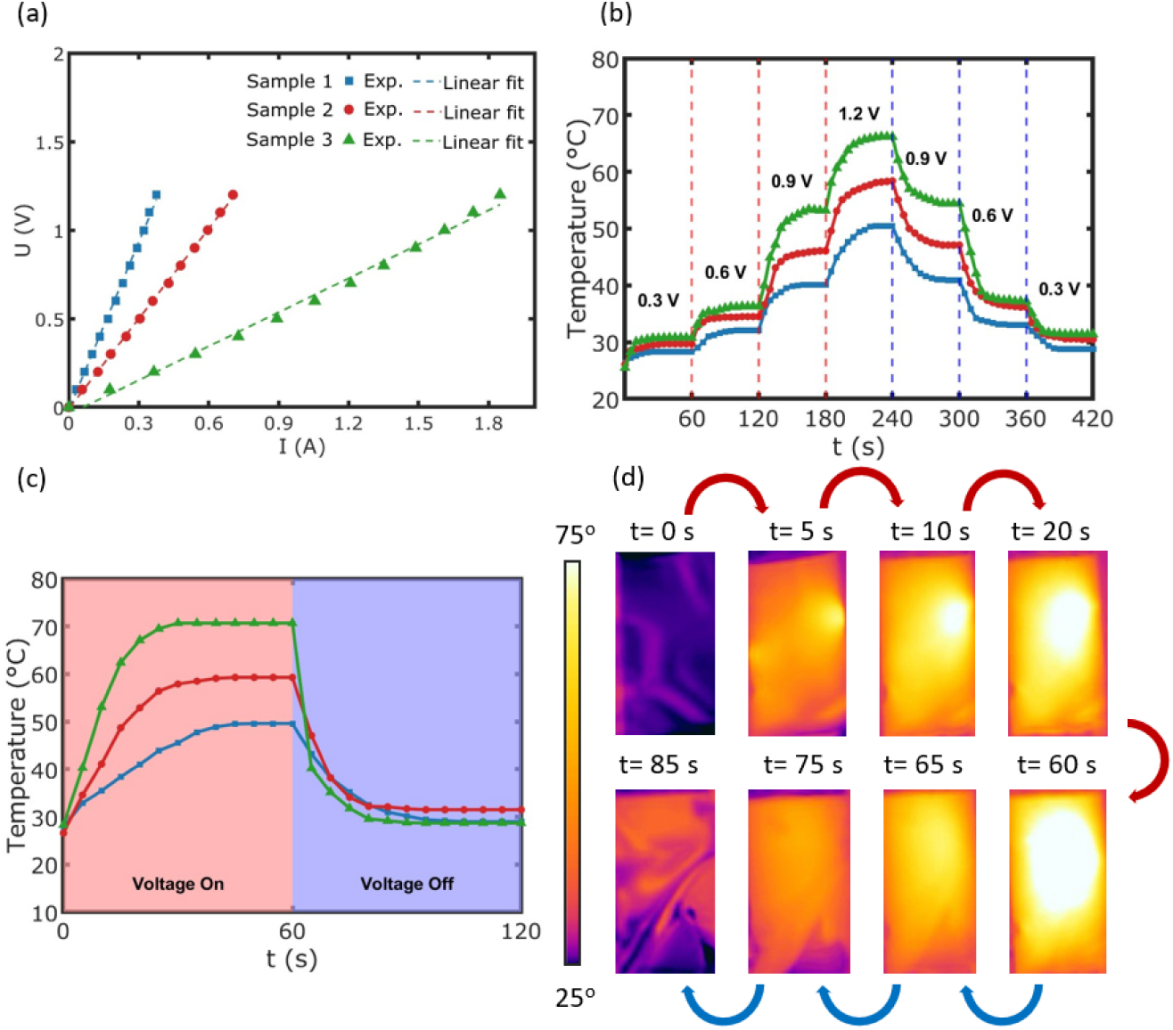
Joule heating performance of flexible metal mesh samples 1, 2, and 3: (a) U–I curve, (b) stepwise heating and cooling curve, (c) Joule heating and cooling curve at 1.2 V, and (d) heat/cooling mapping of sample 3 at 1.2 V during the heating process (indicated by red arrows) and cooling process (indicated by blue arrows). Heat mapping is shown at *t* = 5, 10, 20, and 60 s, while cooling mapping is presented at *t* = 65, 75, and 85 s.

The Joule heating performance of flexible metal mesh conductors embedded in PET is examined, as these structures exhibit both high electrical conductivity and optical transparency. As shown in Figure 7a, the experimental data points for the three metal mesh samples are plotted alongside their respective linear fits. By comparing the experimental data with the linear regression lines, we observe a clear linear relationship between the applied voltage (*U*) and the current (*I*). This linearity is characteristic of Ohmic behavior, indicating that the metal meshes follow Ohm’s law (*I* = *V/R*), where the current is directly proportional to the applied voltage. The close agreement between the experimental data points and their corresponding linear fits further supports this conclusion. Furthermore, the slope of each U–I curve is related to the sheet resistance. Sample 3 with the lowest sheet resistance has the lowest slope, meaning it allows higher current flow at the same voltage compared to samples with higher sheet resistance. Figure 7b shows the stepwise heating and cooling curves for the three samples, evaluating their heating and cooling stability. The temperature data was captured using an IR camera while the heating film was suspended in air, with a coverage area of 1.5 × 3 cm^2^. The voltage was increased in steps of 0.3 V, and the experiments were conducted at four voltage levels: 0.3, 0.6, 0.9, and 1.2 V. For each step, the system was allowed to stabilize for 60 s before the voltage was increased. As observed, samples with lower sheet resistance exhibited greater heating, resulting in higher temperatures. This is consistent with Joule’s law, which states that lower resistance enhances heat generation efficiency at a fixed applied voltage, as more electrical energy is converted into thermal energy. It should be noted that increasing the applied voltage generally leads to higher temperatures. While our study focused on achieving efficient heating at low voltages using low sheet resistance metal meshes, exploring higher voltages was beyond our scope. However, in practical applications where higher temperatures are required, applying greater voltages would likely result in increased heating, following Joule’s law. As depicted in Figure 7c, when a constant voltage of 1.2 V is applied, samples 1, 2, and 3 transition from their initial temperatures of 27.8 °C, 26.6 °C, and 28.3 °C to their respective saturation temperatures of 49.6 °C, 59.3 °C, and 70.7 °C. This temperature increase occurs rapidly. Notably, samples with lower sheet resistance reach their saturation temperature more quickly, indicating enhanced thermal response efficiency. The rapid temperature response to the applied voltage is illustrated for sample 3 through IR images in Figure 7d, captured at different heating (indicated by red arrows) and cooling (indicated by blue arrows) step times. The observed nonuniformities in temperature distribution in Figure 7d arise from sample flexibility, which can cause wrinkles affecting heat dissipation, and from the localized current injection via clamps, leading to variations in heating. Due to their low resistance, metal mesh heaters can generate sufficient heat at lower voltages, improving energy efficiency while maintaining operational safety. The integration of metal mesh into PET substrates enables lightweight, durable, and flexible heating devices, with potential applications in defogging systems, thermotherapy, and wearable heating solutions.

## Conclusion

In conclusion, we have demonstrated a novel and versatile method for fabricating substrate-embedded metal meshes on PET and glass substrates, providing exceptional performance as TCEs and EMI shielding materials. By leveraging plasma treatment, ICP-RIE, and electron-beam evaporation, our approach achieves significant improvements in sheet resistance and durability compared to traditional NSL methods. The fabricated Ag meshes exhibit high FoM and achieves record-breaking performance in terms of FoM and sheet resistance, making them suitable for a wide range of optoelectronic applications.

Furthermore, the embedded design ensures superior adhesion and mechanical durability under bending and tape tests, enabling their use in flexible devices. The high haze values achieved make these meshes particularly advantageous for applications such as solar cells and OLEDs, where light scattering and efficiency are critical. The first demonstration of transparent EMI shielding using this type of metal mesh underscores the broad potential of our method in advanced device applications. As a flexible heater, the metal meshes can achieve a saturation temperature exceeding 70 °C within 60 s under an applied voltage of 1.2 V

This study establishes a foundation for further exploration of NSL-based techniques, with the flexibility to adapt microsphere sizes for tailored optical and electrical properties. Our findings open new avenues for the development of high-performance, durable, and transparent conductive materials for next-generation optoelectronic devices.

## Experimental Section

### Monolayer Formation of PS Microspheres on the Substrate

The fabrication process for forming a hexagonal monolayer of polystyrene (PS) microsphere arrays through self-assembly at the air/water interface is illustrated in Figure S1. PET sheets (MELINEX ST505) having a thickness of 125 μm were purchased from Tekra Inc. The sheets were cut into 9 cm × 8 cm samples using scissors and subjected to ultrasonic cleaning in acetone, methanol, and isopropyl alcohol (IPA) for 10 min each. After cleaning, the samples were dried using nitrogen gas. To make the PET surface hydrophilic, a 20 nm layer of SiO_2_ was sputtered using a physical vapor deposition Angstrom Engineering Sputtering System. Fused silica glass substrates (3 cm × 3 cm) were purchased from UniversityWafer and underwent the same cleaning process as the PET samples. A thick glass slide was placed at the bottom of a 10 cm × 15 cm Petri dish, and the substrate was positioned on top of the glass slide. The Petri dish was then filled with deionized (DI) water until the water level surpassed the substrate’s top surface. A clean, tilted glass slide, treated with IPA to enhance hydrophilicity, was placed in the Petri dish. Three μm PS microspheres were purchased from Polysciences, Inc. The microsphere solution was mixed with ethanol in a 1:1 ratio. Ethanol reduces surface tension, facilitating the more effective spreading of PS nanospheres on the water surface. This mixture was then sonicated for several minutes and, using a pipet, gently applied to the tilted glass slide. The microspheres slid down the tilted glass slide, spreading across the air/water interface. The speed of injecting microspheres must be carefully controlled, as excessive speed can disrupt the dynamic equilibrium of the interface, leading to the sedimentation of PS colloids due to overinjection. The microspheres were added until resistance was observed, indicating saturation at the interface. This resistance is noticeable when additional microspheres begin to sink rather than spread. It is crucial to add enough microspheres to ensure the substrate is fully covered; insufficient microspheres may lead to incomplete coverage. After achieving a saturated interface, the tilted glass slide was removed. To ensure a close-packed microsphere monolayer, 10 wt % sodium dodecyl sulfate (SDS) was used as a surfactant. The microspheres were transferred to the substrate by draining the water through a pipet, leaving the microspheres settled on the substrate. The monolayer was then dried in air at room temperature. As the solvent naturally evaporated, capillary forces bring the nanospheres closer together, resulting in a hexagonally close-packed arrangement that adheres firmly to the substrate.

### Fabrication of Substrate-Embedded Ag Mesh

The fabrication process of substrate-embedded Ag meshes is illustrated in Figure 1. After forming a monolayer of PS microspheres, as detailed in the previous section, ICP-RIE was used for plasma treatment to reduce the size of the microspheres. Plasma treatment for all samples was conducted with an ICP power of 100 W, a bias power of 100 W, a pressure of 30 mT, and an O_2_ flow rate of 50 sccm. The plasma treatment times for Samples 1, 2, and 3, which were fabricated on PET, were set to 80, 105, and 120 s, respectively. For Samples 4 and 5, fabricated on glass, the plasma treatment times were set to 80 and 90 s, respectively. To transfer the gaps between the microspheres into the PET substrate, ICP-RIE was performed with an ICP power of 350 W, a bias power of 100 W, a pressure of 30 mT, a CF_4_ flow rate of 50 sccm, and an SF_6_ flow rate of 20 sccm. The choice of gases for etching PET and glass was based on our previous works.^31,32,36,68^ For glass substrates, an ICP power of 250 W, a bias power of 100 W, a pressure of 30 mT, an Ar flow rate of 50 sccm, and a CHF_3_ flow rate of 25 sccm were employed. Next, silver (Ag) was deposited onto the substrate using a Plassys E-Beam Evaporation System. E-beam evaporation was chosen because it provides anisotropic deposition, unlike sputtering, which deposits materials isotropically. Finally, PS microspheres were removed by a liftoff process, revealing the final substrate-embedded Ag mesh.

### Characterization

A probe station equipped with a semiconductor device analyzer (B1500A Semiconductor Device Analyzer, Keysight Technologies) utilizing the four-needle Van der Pauw method was used to measure sheet resistance. High-resolution imaging of the glass-embedded Ag micromesh was conducted using scanning electron microscopy (Zeiss SIGMA VP). Total and direct transmittance in the wavelength range of 400–800 nm were measured with a UV–vis-NIR spectrometer featuring a 100 mm diameter integrating sphere and a 2D detector (PerkinElmer Lambda 750). The transmission values reported in this study were calculated by normalizing the measured transmission data to exclude the contribution of bare PET or glass. This was achieved by dividing the measured transmission values by the transmission of the bare PET or glass substrate. EMI SE was determined using the coaxial transmission line method with an HP 7822D Vector Network Analyzer (VNA) for signal generation and detection. The sample was placed between two waveguide flanges, selected based on the target frequency range. The waveguide flanges were securely fastened using screws and nuts to prevent movement during measurements. The X-band and Ku-band waveguide flanges were sourced from PASTERNACK. For Joule heating, a DC source (NANKADF 3A V 1AA bench power supply) was used, with alligator clips to connect to the flexible metal mesh samples. The temperature during operation was monitored using a FLIR ONE PRO infrared camera.

## Supporting Information Available

Schematic illustration of the process for forming a monolayer of microspheres on a substrate; SEM images of 3 μm PS microspheres after ICP-RIE with different ICP powers; SEM images of the PET substrate after removing the microspheres using ICP-RIE; comparison table of the performance of our metal meshes with other metal meshes reported in the literature fabricated by nanosphere lithography as transparent electrodes; transmission and haze plots for Samples 6–8; EMI Shielding Measurements formulas (PDF)
