# Supplementary material for: Flexible Embedded Metal Meshes by Nanosphere Lithography for Very Low Sheet Resistance Transparent Electrodes, Joule Heating, and Electromagnetic Interference Shielding
Source: ACS Appl Electron Mater. 2025 Apr 28;7(9):4266–78. doi: 10.1021/acsaelm.5c00425 (PMC12080244; doi:10.1021/acsaelm.5c00425)
Supplement: Supplementary file 1 — el5c00425_si_001.pdf [file el5c00425_si_001.pdf]

Supporting Information
Flexible Embedded Metal Meshes by Nanosphere Lithography for Very Low Sheet Resistance Transparent Electrodes, Joule Heating, and Electromagnetic Interference Shielding

Mehdi Zarei,[†] Khashayar Mohammadi,[‡] Abdullah A. Mahmood,[¶] Mingxuan Li,[§] and Paul W Leu[*,‖,†,§]

†*Department of Mechanical Engineering, University of Pittsburgh, Pittsburgh, PA 15261, United States*

‡*Department of Civil Engineering, University of Waterloo, Waterloo, N2L3G1, Canada*

¶*Department of Electrical and Computer Engineering, University of Pittsburgh, Pittsburgh, PA 15261, United States*

§*Department of Chemical Engineering, University of Pittsburgh, Pittsburgh, PA 15261, United States*

‖*Department of Industrial Engineering, University of Pittsburgh, Pittsburgh, PA 15261, United States*

E-mail: pleu@pitt.edu

Figure S1 illustrates the schematic process of forming a monolayer of polystyrene (PS)

microspheres (MS) on a substrate. Here, we employ a self-assembly approach at the liquid–air interface which, compared to other methods such as drop-casting, drag coating, electrostatic adsorption, dip coating, rub coating, spin coating, and confined convective assembly, yields higher-quality films while allowing greater flexibility in substrate selection.[1] A glass slide with the substrate on top is placed in a Petri dish filled with deionized (DI) water, with the water level extending above the substrate's top surface. A solution of MS is then injected onto the tilted glass slide, causing the MS to spread at the water/air interface. The Marangoni effect, driven by surface tension gradients, serves as the driving force for the spreading of PS microspheres by inducing mass transfer along the interface between two fluids. Next, a sodium dodecyl sulfate (SDS) surfactant is added, resulting in a close-packed hexagonal array of MS at the interface. Finally, the water is drained using a pipette, allowing the MS to settle onto the substrate. In (d), a homogeneous monolayer of 3 μm PS microspheres is visible on a 9 cm $\times$ 8 cm PET substrate after all water is drained from a 10 $\times$ 15 cm Petri dish. Some bubbles are visible in the image, which are attributed to water that has moved beneath the PET substrate. However, these bubbles do not affect the quality of the microsphere monolayer formation on the top surface. This process is easily scalable to any desired PET or glass substrate size by selecting Petri dishes and substrates of appropriate dimensions.

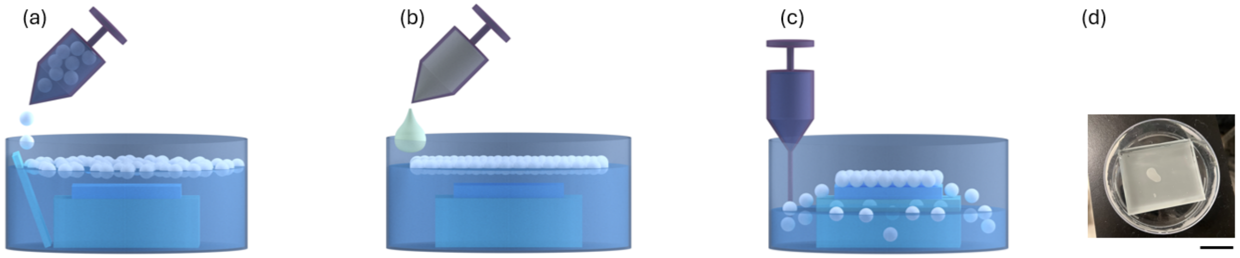

Figure S1: Schematic illustrating the process of forming a monolayer of microspheres on a substrate: (a) Injection of the microsphere solution onto a tilted glass slide, resulting in the random dispersion of microspheres at the water/air interface; (b) Addition of an SDS surfactant solution, aligning the microspheres into a close-packed hexagonal structure; (c) Gradual water drainage to lower the water level, enabling the microspheres to settle onto the substrate; (d) Formation of a uniform monolayer of 3 μm PS microspheres on an 9 cm × 8 cm PET substrate after complete water drainage from a 10 × 15 cm Petri dish. The scale bar represents 3 cm. Some air bubbles can be seen under the substrate.

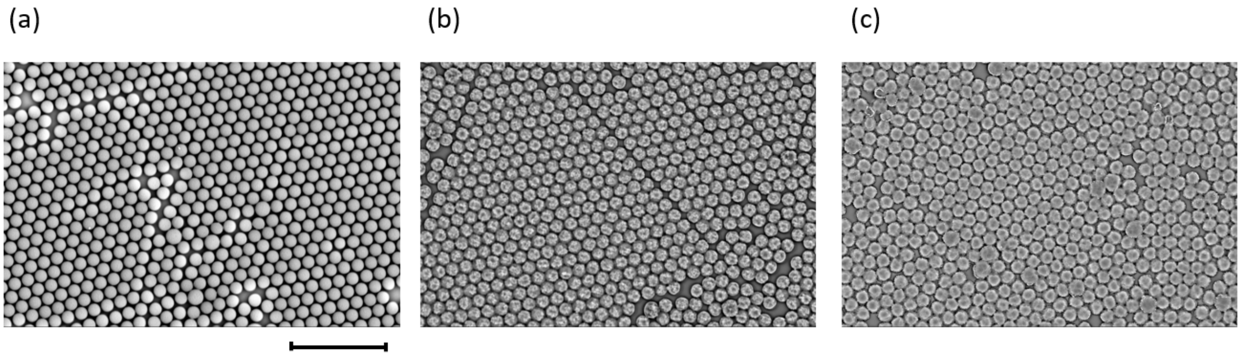

Figure S2: Top-view SEM images of 3 μm PS microspheres on a glass substrate after ICP-RIE with ICP powers of (a) 250 W, (b) 400 W, and (c) 600 W. The other plasma treatment and ICP-RIE parameters are consistent with those used for samples 4 and 5. All samples underwent 100 seconds of plasma treatment, and the ICP-RIE duration was 7 minutes for all three samples. The scale bar represents 20 μm.

Figure S2 shows top-view SEM images of 3 μm PS microspheres on a glass substrate after ICP-RIE treatment with ICP powers of (a) 250 W, (b) 400 W, and (c) 600 W, with all other plasma treatment and ICP-RIE parameters consistent with those used for samples 4 and 5. All samples underwent 100 seconds of plasma treatment, and the ICP-RIE duration was 7 minutes for all three samples. As observed, at an ICP power of 250 W, the microspheres exhibited very good tolerance against ICP-RIE, with minimal effects. However, at ICP powers of 400 W and 600 W, both the microspheres and the substrate were significantly

affected by etching, leading to reduced control over the etching process. Since deposition is intended to occur after ICP-RIE, the damage to the microspheres makes their presence as a mask unfeasible. Additionally, substrate etching introduces surface roughness.

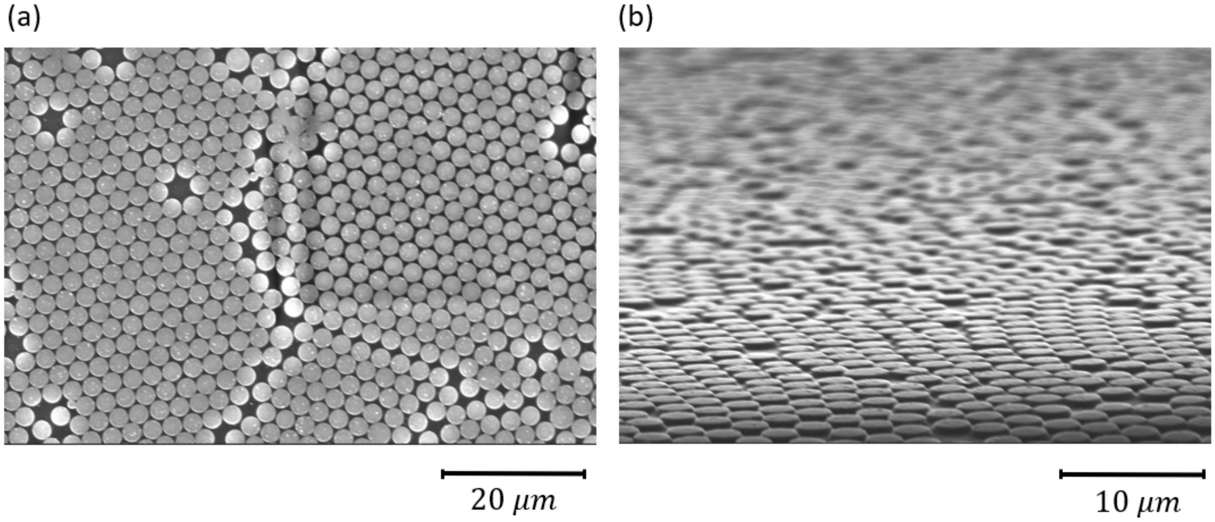

Figure S3: SEM images of the PET substrate after removing the microspheres using ICP-RIE: (a) Top-view and (b) side-view taken at an 85° angle from the normal direction to the substrate, providing an almost parallel perspective.

Table S1: Comparison of the performance of our metal meshes with other nanosphere lithography metal meshes in the literature as transparent electrodes

Literature	Substrate	T (%)	$R_s(\Omega/sq)$	σ_{DC}/σ_{OP}
This work	PET	73.1	1.52	737
This work	PET	64.3	0.51	1492
This work	PET	58.1	0.22	2736
This work	Glass	75.1	1.61	756
This work	Glass	65.6	0.38	2113
Gao *et. al*[2]	PET	80.0	60.5	26.0
Gao *et. al*[2]	Glass	80.0	17	94
Qiu *et. al*[3]	PET	90.0	62.0	56.0
Qiu *et. al*[3]	Glass	88.4	30	99.0
Wang *et. al*[4]	PET	81	20.9	81
Torrisi *et. al*[5]	Glass	66.0	12.8	64
Wu *et. al*[6]	Glass	89.2	16.9	190.2
Qiu *et. al*[7]	Glass	55.0	5.2	104
Bley *et. al*[8]	Glass	81.0	41.4	41.0
Kim *et. al*[9]	Glass	45.7	3.7	106.3

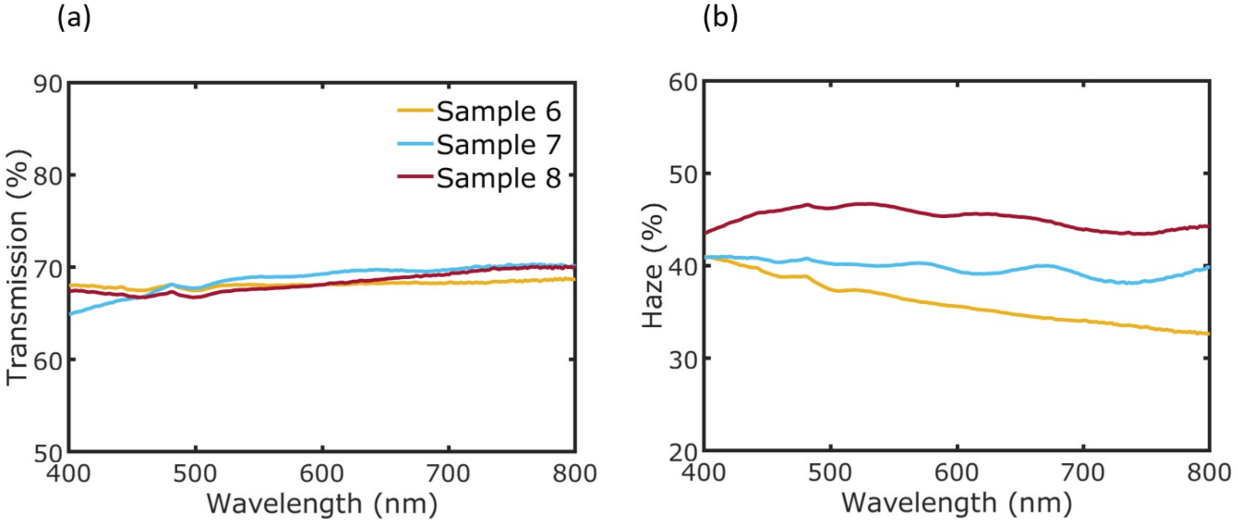

Figure S4: (a) Transmission and (b) haze as a function of wavelength within the visible spectrum for samples 6, 7, and 8.

Figure S4 shows (a) transmission and (b) haze as a function of wavelength within the visible spectrum for samples 6, 7, and 8. The plasma treatment times for these samples are 140, 120, and 80 seconds, respectively. The ICP-RIE times are 3.5, 9, and 20 minutes, respectively, resulting in estimated depths of 150 nm, 400 nm, and 900 nm, respectively. These samples have transmission of 68.1%, 69.0%, and 67.6%, respectively.

EMI Shielding Measurements

We use the waveguide approach for analyzing the EMI shielding performance of various materials. In this method, the experimental setup for the vector network analyzer (VNA) waveguide involves sandwiching the test sample between two flanges, which are then connected to the waveguides of the VNA.[10] During each test, the VNA transmits an EM wave from port 1 at a specific frequency, while the detector records the corresponding transmitted (S_{21}) and reflected (S_{11}) signals. Similarly, when the signal is sent from port 2, the detector captures S_{22} and S_{12} parameters. As the frequency of the source changes, new measurements are taken accordingly. The results of these tests are represented in terms of complex

scattering (S) parameters, which describe the reflection and transmission characteristics of the material over a specific frequency range.

Material uniformity is a key factor in these measurements, ensuring that $S_{11} = S_{22}$ and $S_{21} = S_{12}$, thereby reinforcing the consistency of the results. The derived S-parameters provide a comprehensive understanding of the material's shielding properties, which can be further analyzed using transmission line theory to determine three critical EMI shielding coefficients: reflectance (R_{rf}), transmittance (T_{rf}), and absorbance (A_{rf}).

These parameters are mathematically defined as follows:

$$R_{rf} = \frac{P_R}{P_I} = |S_{11}|^2 = |S_{22}|^2 \tag{S1}$$

$$T_{rf} = \frac{P_T}{P_I} = |S_{21}|^2 = |S_{12}|^2 \tag{S2}$$

$$A_{rf} = 1 - R_{rf} - T_{rf} \tag{S3}$$

Using these coefficients, the total shielding effectiveness (SE_T), reflection shielding effectiveness (SE_R), and absorption shielding effectiveness (SE_A) can be determined as:

$$SE_T = 10\log_{10}\left(\frac{1}{T_{rf}}\right) = -10\log_{10}|S_{12}|^2 \tag{S4}$$

$$SE_R = 10\log_{10}\left(\frac{1}{1 - R_{rf}}\right) = -10\log_{10}(1 - |S_{11}|^2) \tag{S5}$$

$$SE_A = 10\log_{10}\left(\frac{1 - R_{rf}}{T_{rf}}\right) = 10\log_{10}\left(\frac{1 - |S_{11}|^2}{|S_{12}|^2}\right) \tag{S6}$$

These relationships provide a quantitative assessment of EMI shielding performance, enabling the evaluation of a material's effectiveness in attenuating electromagnetic waves.

References

(1) Gao, P.; He, J.; Zhou, S.; Yang, X.; Li, S.; Sheng, J.; Wang, D.; Yu, T.; Ye, J.; Cui, Y. Large-Area Nanosphere Self-Assembly by a Micro-Propulsive Injection Method for High Throughput Periodic Surface Nanotexturing. *Nano Letters* **2015**, *15*, 4591–4598.

(2) Gao, T.; Wang, B.; Ding, B.; Lee, J.-K.; Leu, P. W. Uniform and Ordered Copper Nanomeshes by Microsphere Lithography for Transparent Electrodes. *Nano Letters* **2014**, *14*, 2105–2110.

(3) Qiu, T.; Luo, B.; Akinoglu, E. M.; Yun, J.; Gentle, I. R.; Wang, L. Trilayer Nanomesh Films with Tunable Wettability as Highly Transparent, Flexible, and Recyclable Electrodes. *Advanced Functional Materials* **2020**, *30*, 2002556.

(4) Wang, S.; Lv, L.; Wang, H. An extraordinary colloidal lithographic template: CaCO3 micro-hemisphere arrays applied in fabricating Cu mesh transparent conductive film. *Applied Surface Science* **2024**, *665*, 160353.

(5) Torrisi, G.; Luis, J. S.; Sanchez-Sobrado, O.; Raciti, R.; Mendes, M. J.; Águas, H.; Fortunato, E.; Martins, R.; Terrasi, A. Colloidal-structured metallic micro-grids: High performance transparent electrodes in the red and infrared range. *Solar Energy Materials and Solar Cells* **2019**, *197*, 7–12.

(6) Wu, S.; Cossio, G.; Braun, B.; Wu, F. C. M.; Yu, E. T. Smart Window Structures Based on Highly Conductive, Transparent Metal Nanomeshes and Thermochromic Perovskite Films. *Advanced Optical Materials* **2023**, *11*, 2202409.

(7) Qiu, T.; Luo, B.; Ali, F.; Jaatinen, E.; Wang, L.; Wang, H. Metallic Nanomesh with Disordered Dual-Size Apertures As Wide-Viewing-Angle Transparent Conductive Electrode. *ACS Applied Materials & Interfaces* **2016**, *8*, 22768–22773.

(8) Bley, K.; Semmler, J.; Rey, M.; Zhao, C.; Martic, N.; Klupp Taylor, R. N.; Stingl, M.; Vogel, N. Hierarchical Design of Metal Micro/Nanohole Array Films Optimizes Transparency and Haze Factor. *Advanced Functional Materials* **2018**, *28*, 1706965.

(9) Kim, Y.; Jang, E.; Lee, Y.; Oh, C.; Kim, K.; Kook, G.; Kim, M. K.; Lee, M.; Lee, H. J. Miniature Transparent Dopamine Sensor Based on Nanosphere Lithography. *Advanced Materials Technologies* **2023**, *8*, 2300006.

(10) Isari, A. A.; Ghaffarkhah, A.; Hashemi, S. A.; Wuttke, S.; Arjmand, M. Structural Design for EMI Shielding: From Underlying Mechanisms to Common Pitfalls. *Advanced Materials* **2024**, *36*, 2310683.
